# The Influence of Technological Factors on the Structure and Chemical Composition of Tuberous *Dahlia* Roots Determined Using Vibrational Spectroscopy

**DOI:** 10.3390/plants13141955

**Published:** 2024-07-17

**Authors:** Ioana Moldovan, Alex-Péter Cotoz, Sándor Rózsa, Klara Magyari, Lukács Lehel, Monica Baia, Maria Cantor

**Affiliations:** 1 Horticultural Research Station, University of Agricultural Sciences and Veterinary Medicine Cluj-Napoca, 400372 Cluj-Napoca, Romania; ioana.moldovan@usamvcluj.ro (I.M.); lehel.lukacs@usamvcluj.ro (L.L.); 2 Department of Horticulture and Landscape Design, University of Agricultural Sciences and Veterinary Medicine Cluj-Napoca, 400372 Cluj-Napoca, Romania; alex.cotoz@usamvcluj.ro (A.-P.C.); rozsa.sandor@usamvcluj.ro (S.R.); 3 Institute for Interdisciplinary Research, Bio-Nano-Sciences Babes-Bolyai University, Treboniu Laurean 42, 400271 Cluj-Napoca, Romania; klara.magyari@ubbcluj.ro; 4 Faculty of Physics, Babes-Bolyai University, M. Kogâlniceanu 1, 400084 Cluj-Napoca, Romania

**Keywords:** *Dahlia*, inulin content, lignin content, suberin content, FT-IR imaging

## Abstract

This research investigated the structural and chemical modifications of *Dahlia* ‘Kennemerland’ across different technological conditions and throughout the vegetation period. Using FT-IR imaging, this study focused on the changes in the inulin, lignin, and suberin contents of tuberous roots. FT-IR maps were generated to visualize the distribution of these compounds across scanned areas, highlighting variations across cultivation methods and seasonal stages. The key compounds analyzed included inulin, lignin, and suberin, which were identified in different root zones. The results showed that inulin was distributed in all analyzed areas, predominantly in zone 1 (periderm), with a distribution that increased with forced cultivation, while lignin and suberin distributions varied with zone and season. Forced tuberous root lignin was detected in all four areas analyzed, in the fall accumulating mainly in area 4 and in suberin starting from summer until autumn. Based on the evaluation of the maps obtained by representing the area ratios of specific bands (inulin/lignin and inulin/suberin), we established where the inulin was present in the highest quantity and concluded that suberin was the constituent with the lowest concentration in tuberous *Dahlia* roots. These findings emphasize the influence of technological factors and seasonal changes on the biochemical makeup of tuberous *Dahlia* roots. This detailed biochemical mapping provides insights for optimizing *Dahlia* cultivation and storage for various industrial applications. This study concludes that FT-IR spectroscopy is an effective tool for monitoring and understanding the biochemical dynamics of *Dahlia* roots, aiding their agricultural and industrial utilization.

## 1. Introduction

*Dahlia hybrida*, an ornamental species of significant economic importance, is known for its adaptability to various environmental conditions. It finds applications in pharmaceuticals, cosmetics, food, and dye extraction. During the summer–autumn season, *Dahlia* is prominent in the cut flower market, yielding 15 to 20 flower stems per plant, depending on the variety [1]. The genus *Dahlia* comprises around 30 perennial species with tuberous roots within the Asteraceae family [2] and includes approximately 50,000 cultivated varieties worldwide [3]. While *Dahlias* thrive in frost-free climates, their tuberous roots can endure dormancy periods, allowing cultivation in temperate climates via the lifting and storing of the tubers during winter [4]. In Romania, *Dahlia* spp. do not survive winter if left in gardens during severe frosts or wet conditions but add late summer color to borders from July to October [5].

*Dahlia* tubers are valuable for their inulin content, used in medicine and the food industry [6,7,8]. The inulin present in *Dahlia* tubers [9] is found in both monocotyledonous and dicotyledonous families, predominantly in the Asteraceae, Boraginaceae, Asparagus [10], Liliaceae, and Amaryllidaceae [11]. The tuberous roots contain carbohydrates, mainly inulin, in the inner cortex and parenchyma layers [12]. Researchers have identified inulin in various tissues of Asteraceae roots, describing it as crystals resembling tiny Maltese crosses under polarized light [13,14]. Inulin, a polymer of fructose, is of interest owing to its bioactivity and applications in medicine and food [15]. It is a prebiotic not digestible by human enzymes [16] and is well tolerated as a food ingredient [7].

The inulin quantity in plants varies with species, natural conditions, and maintenance [17]. The inulin in *Dahlia* tubers, which is composed of 35 fructose molecules linked by *β*-2,1-glycosidic bonds with a glucose molecule at the end [18], acts as a substitute for fat and sugar and serves as a dietary fiber [2,19]. It offers low-calorie benefits and is recognized for its prebiotic properties [7,20,21]. In pharmaceutical applications, inulin is used as a stabilizer, excipient, or injectable [19,22]. It also has significant biological effects, such as immune system stimulation and anticarcinogenic properties [9,10,22]. In summary, inulin has been shown to positively impact gastrointestinal health, alleviate constipation, enhance mineral absorption, and regulate glycemic levels.

In the chemical industry, inulin is used for alcohol production, including ethanol, as a sugar source, and as a precursor in producing chemicals like glycerol [23]. Its use in processed foods is growing due to its benefits, including suitability for people with diabetes [24]. Inulin’s molecular characteristics vary with plant source, climate, growing conditions, harvesting maturity, and storage time [25].

By examining *Richterago* sp., another Asteraceae member, researchers observed inulin alongside other compounds like lignin, cellulose, hemicellulose, and pectin in the tubers [13]. Suberin is a hydrophobic substance present in the cell walls of plants. It plays an essential role in water regulation, controlling its movement within plants, and is vital in roots through the formation of the Casparian strip. Additionally, suberin provides protection, acting as a barrier against pathogens and physical damage [26].

Lignin is a complex and rigid polymer present in the secondary cell wall. It offers structural support, ensuring rigidity and support to plants, and is essential in water transport, strengthening xylem vessels and preventing their collapse. Furthermore, lignin protects plants against pathogens and decomposition [26,27,28].

Infrared (IR) and Raman spectroscopy are key techniques for analyzing molecular vibrations, with IR detecting absorption and Raman detecting scattering [29]. These methods have identified and quantified plant substances [30,31,32], and future advances may integrate vibrational spectroscopy mapping for detailed analyses [33]. This study aimed to obtain precise biochemical composition data of *Dahlia* ‘Kennemerland’ tubers using FT-IR spectroscopy, revealing structural differences and quantitative information on chemical compounds.

This study fills crucial gaps in the research on the biochemical composition of tuberous *Dahlia hybrida* roots by employing advanced FT-IR spectroscopy techniques. It provides detailed mapping of the distribution of inulin, lignin, and suberin contents across various root zones and seasons. By comparing forced and nonforced cultivation methods, this research highlights the effects of these practices on the biochemical composition of the tubers. This study deepens the understanding of *Dahlia* biochemistry, providing information for optimizing its cultivation to improve yield and quality, with significant implications for pharmaceutical applications.

## 2. Results

### 2.1. Biochemical Composition of the Forced and Nonforced Tuberous Roots of Dahlia hybrida during an Entire Vegetative Year

**Figure 1:**
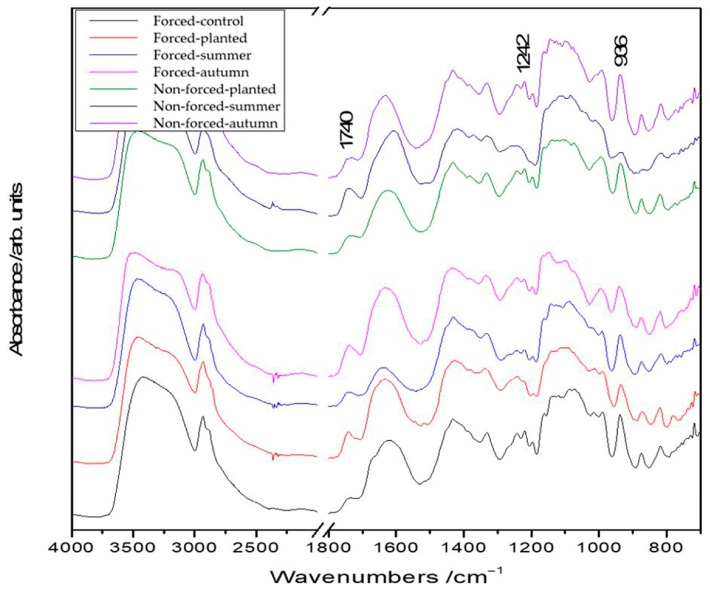
FT-IR spectra for the forced/nonforced tuberous roots of the cultivar ‘Kennemerland’ recorded at the end of vegetative repose and during the entire vegetative stage.

To assess the influence of different technological factors on the biochemical composition of the tuberous roots, FT-IR spectra were recorded and analyzed. Figure 1 presents the FT-IR spectra recorded on a section of the tuberous roots from zone 1 of the cultivar ‘Kennemerland’ at the end of the vegetative repose and throughout the vegetative stage. The assignment of the characteristic bands to the corresponding vibrations are summarized in Table 1. The bands attributed to inulin, lignin, and suberin vibrations were selected to characterize all identified zones in the structure of the tuberous *Dahlia* tuberous roots. Thus, the inulin band was identified at 936 cm^−1^, the lignin band at 1242 cm^−1^ in all four zones, and the suberin band at 1740 cm^−1^ only in zone 1 (periderm).

These bands highlighted the structural differences in the tuberous roots throughout the vegetation period. FT-IR mapping of the ratios of the characteristic bands (936/1242 cm^−1^ and 936/1740 cm^−1^) was used to obtain quantitative information across different zones of the tuberous roots.

### 2.2. FT-IR Mapping Analysis

The results of the biochemical analysis of the tuberous roots of *Dahlia* cultivar ‘Kennemerland’ are illustrated in the subsequent figures. These mappings provide a visual representation of the distribution and concentration of the key biochemical compounds within the roots, offering a comprehensive overview of their variations.

**Figure 2:**
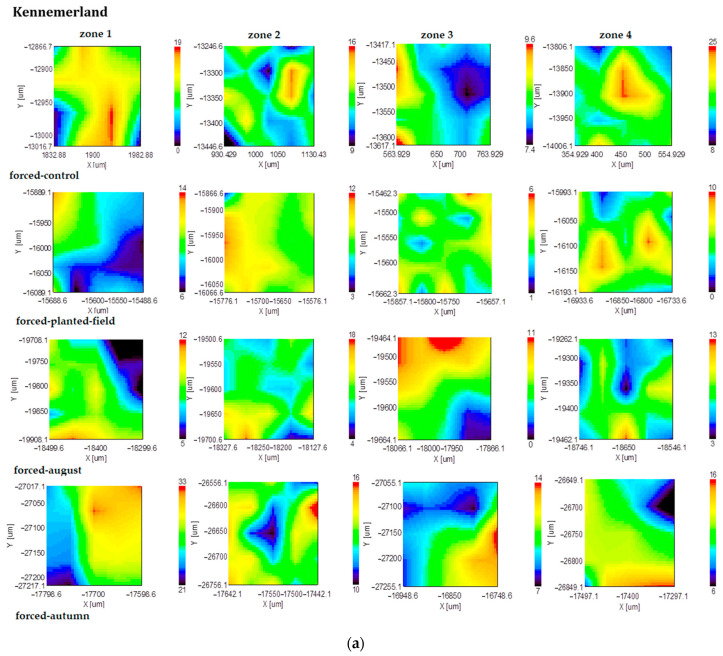

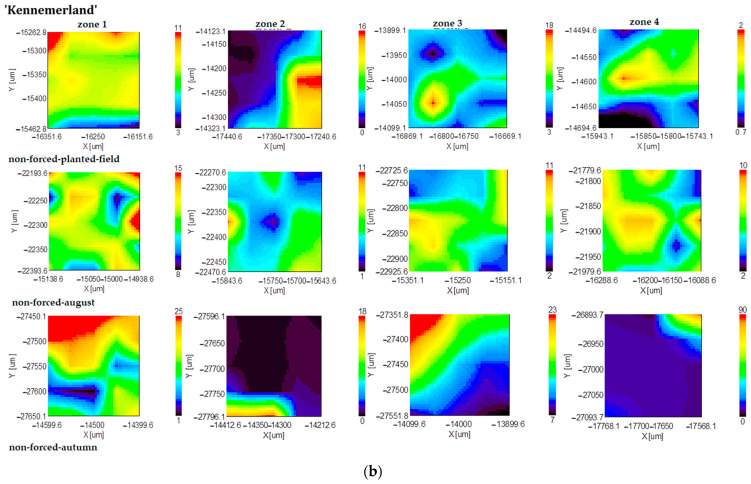
FT-IR mapping of the inulin content (936 cm^−1^ band area) of forced (**a**) and nonforced (**b**) tuberous roots of the cultivar ‘Kennemerland’ recorded at the end of vegetative repose and during the entire vegetative stage.

The mappings obtained from representing the inulin band on the scanned surface area (Figure 2) reveal that despite its unexpected presence in this area, inulin was predominant in zone 1. Forcing induced an intensification of inulin distribution in zones 2, 3, and 4. In August, for nonforced tuberous roots, the distribution of the intensity of the absorption band at 936 cm^−1^ in zone 1 remained stable, while it changed slightly in zones 2, 3, and 4. From the onset of cultivation up until the month of August, the distribution of inulin remained consistent in forced culture. By fall, for forced planting material, inulin was exclusively detected in zone 1, with reduced presence observed in other zones. Conversely, the distribution of inulin for nonforced planting material remained consistent across the observed period.

**Figure 3:**
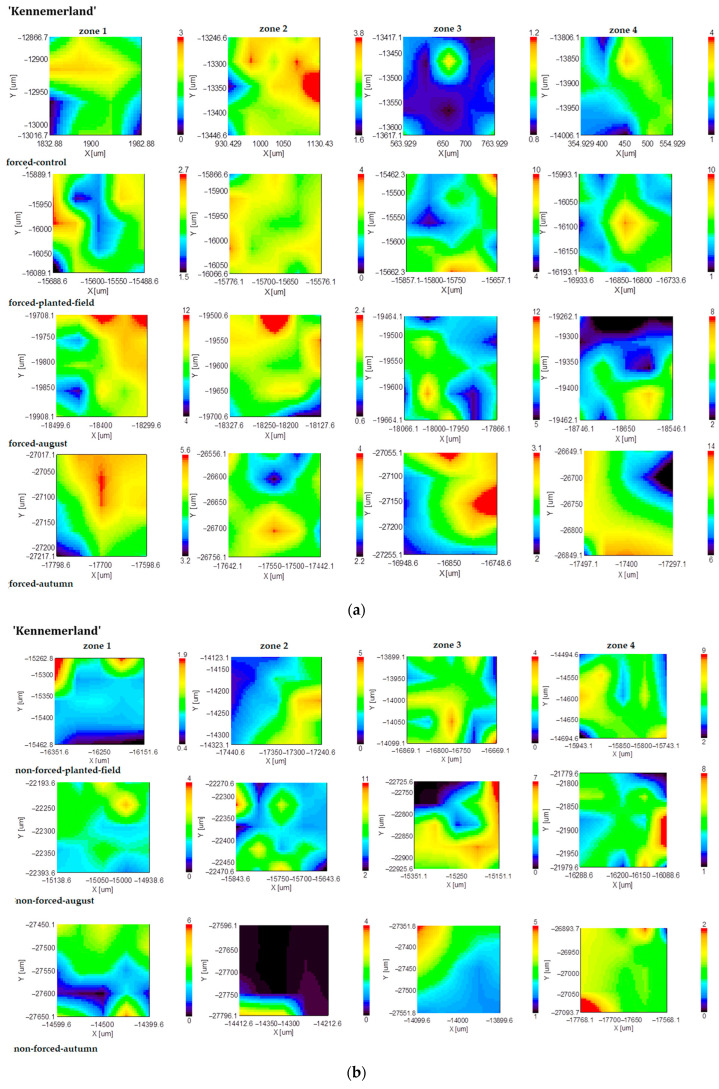
FT-IR mapping of the lignin content (1242 cm^−1^ band area) of forced (**a**) and nonforced (**b**) tuberous roots of the cultivar ‘Kennemerland’ recorded at the end of vegetative repose and during the entire vegetative stage.

Regarding lignin, the mappings obtained for the forced tuberous roots indicate its predominance in zones 1, 2, and 4 (Figure 3). Extended vegetative periods led to a decrease in the lignin distribution in zones 1 and 2, while zones 3 and 4 experienced an increase. In forced tuberous roots initially cultivated in a greenhouse and subsequently planted in the field, the area where lignin was present increased across all zones of the tuberous roots. In August, there were no significant changes observed in the lignin distribution for either forced or nonforced tuberous roots. From August to fall, the lignin band distribution decreased in zone 2 and increased in zone 4 for nonforced planting material, whereas for forced planting material, lignin predominantly accumulated in zone 4 and remained stable in zones 1, 2, and 3.

**Figure 4:**
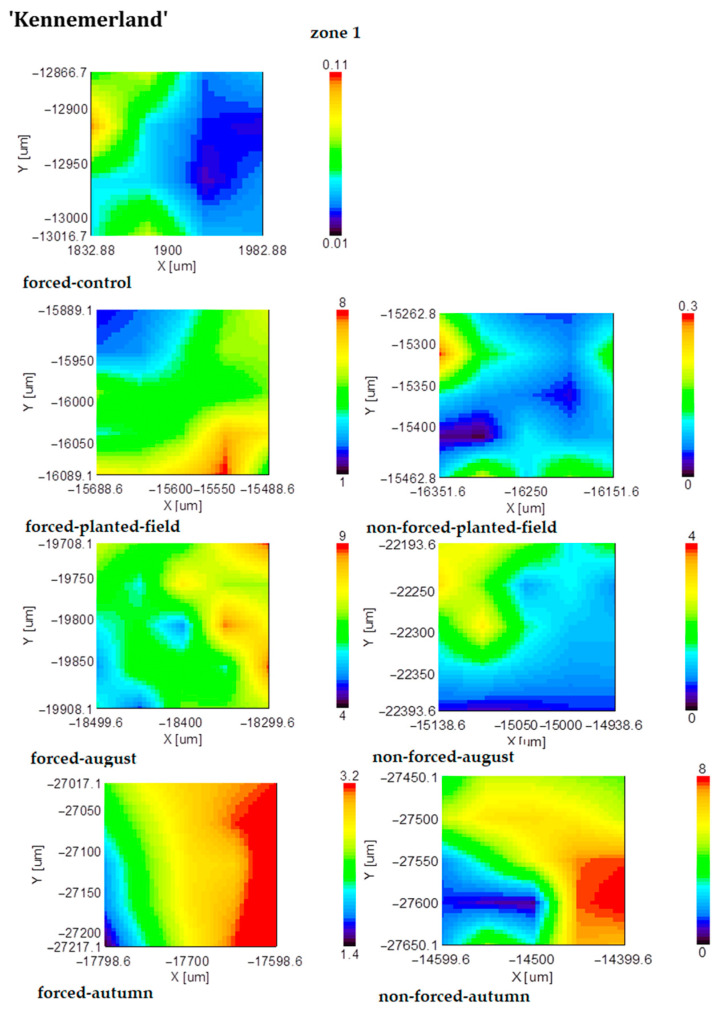
FT-IR mapping of the suberin content (1740 cm^−1^ band area) of forced and nonforced tuberous roots of the cultivar ‘Kennemerland’ recorded at the end of vegetative repose and during the entire vegetative stage.

In nonforced tuberous roots, suberin accumulated in the fall, whereas in forced tuberous roots, it began to accumulate from summer to fall (Figure 4).

**Figure 5:**
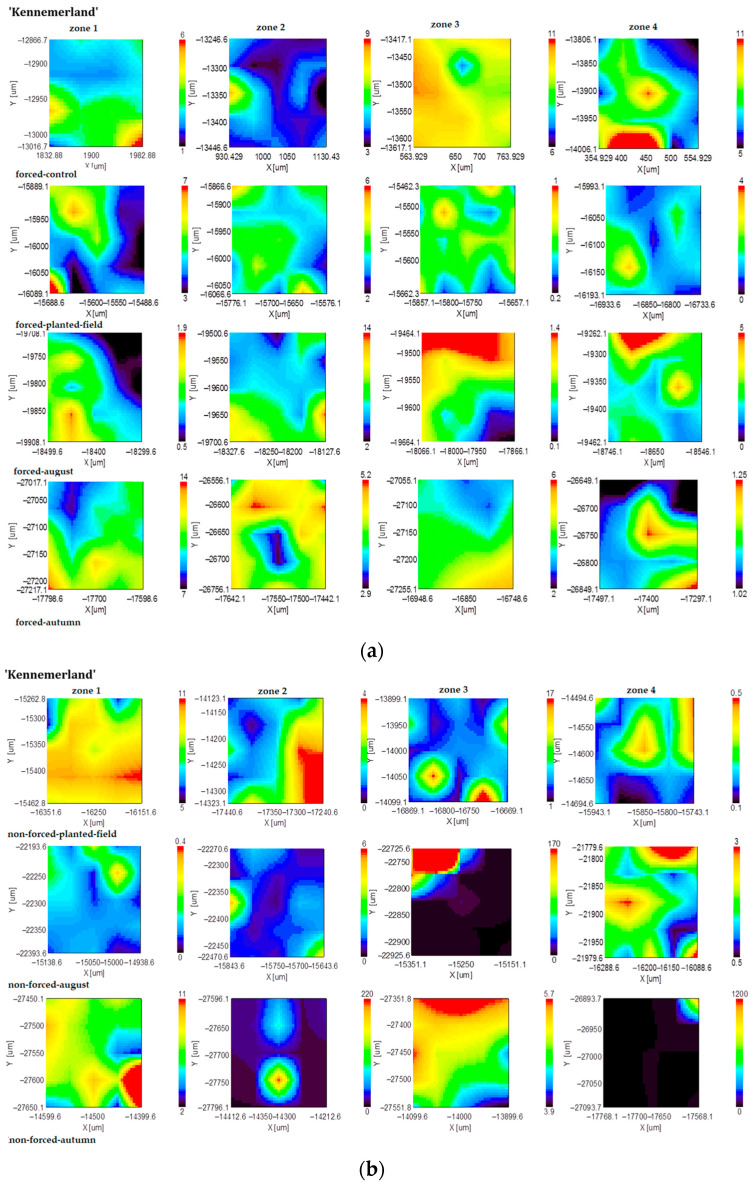
FT-IR mapping of the inulin/lignin ratio (intensity ratio of 936 cm^−1^ and 1242 cm^−1^ bands) of forced (**a**) and nonforced (**b**) tuberous roots of the cultivar ‘Kennemerland’ recorded at the end of vegetative repose and during the entire vegetative stage.

**Figure 6:**
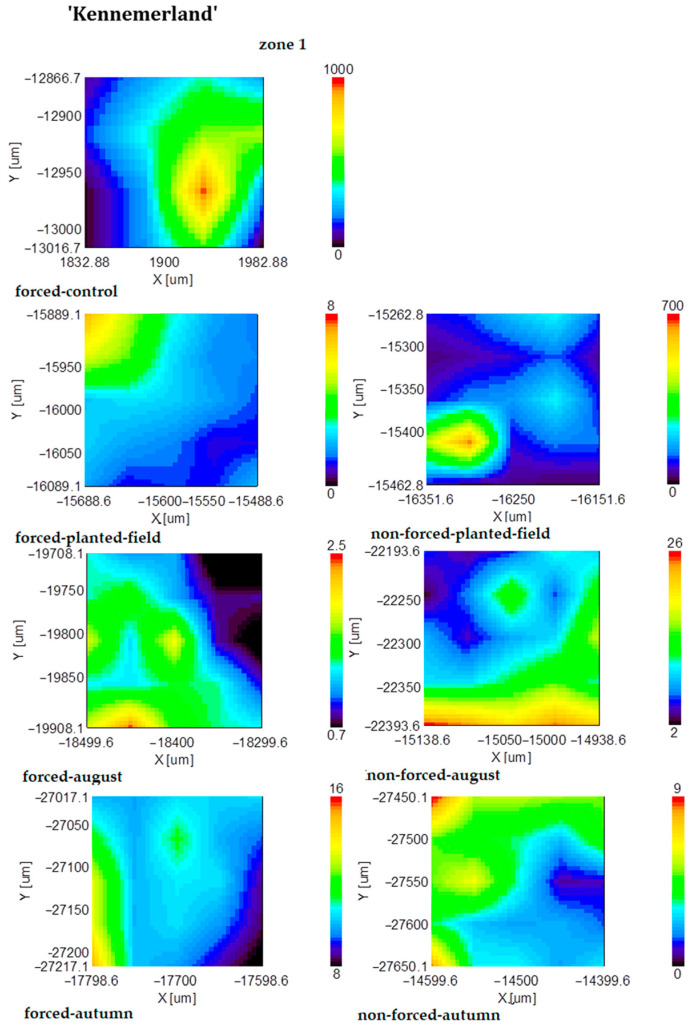
FT-IR mapping of the inulin/suberin ratio (intensity ratio of 936 cm^−1^ and 1740 cm^−1^ bands) of forced and nonforced tuberous roots of the cultivar ‘Kennemerland’ recorded at the end of vegetative repose and during the entire vegetative stage.

In analyzing the mapping representing the distribution of the characteristic bands’ ratios of 936 cm^−1^/1242 cm^−1^ (inulin/lignin) and 936 cm^−1^/1740 cm^−1^ (inulin/suberin) illustrated in Figure 5 and Figure 6, respectively, the following observations were made:

Inulin/lignin ratio: Forced tuberous roots accumulated more lignin than inulin in zone 2, while the ratios were reversed in zone 3. Until planting in the field, nonforced tuberous roots saw increases in inulin in zone 1 and lignin in zone 3. From forcing tuberous roots to field planting, inulin decreased across all zones.

In Figure 5, we can observe how lignin increased in zones 1, 2, and 3 for nonforced tuberous roots from spring to summer, with inulin increasing in zone 4. In contrast, forced roots showed increases in inulin in zones 3 and 4 but decreases in zones 1 and 2. By autumn, nonforced tuberous roots exhibited significant increases in lignin in zones 1, 2, and 4 and inulin in zones 1 and 2. Meanwhile, forced tuberous roots displayed increases in lignin in zones 4 and 3, with the highest accumulation of inulin observed in zone 1.

Inulin/suberin ratio: From the obtained images (Figure 6), it was observed that forced tuberous roots accumulated a large amount of inulin in spring. Upon planting in the field, inulin accumulated only in a restricted area. In August, the amount of inulin decreased in both forced and nonforced tuberous roots.

The amount of suberin was higher in certain regions of the nonforced tuberous roots compared to the forced ones, where it was found in equal amounts.

## 3. Discussion

The collected data revealed significant variability in the chemical compounds (inulin, lignin, suberin) identified in the analyzed samples. These findings are consistent with those reported by other researchers [34,35]. During a 3-month study on the storage of Jerusalem artichoke tubers and the analysis of inulin in various crops and storage conditions, it was observed that the inulin composition remained stable at −18 °C. However, significant degradation of the inulin into sucrose and fructo-oligosaccharides occurred after 4 weeks of storage at 4 °C [19]. Despite the observed differences in the inulin content across treatments, our collected data were in line with those previously reported. Any fluctuations in inulin content could be attributed to seasonal variations.

In previous studies, researchers demonstrated the significance of both suberin and lignin as essential components of the endodermal cell wall of the roots across various monocotyledonous species, such as *Monstera deliciosa* Liebm., *Iris germanica* L., *Allium cepa* L., *Aspidistra elatior* Bl., and *Agapanthus africanus* (L.) Hoffmgg. [36]. Researchers asserted that while the composition of suberin is similar across species, its content varies significantly depending on the species [37]. This variability can be induced by a range of environmental factors, ultimately influencing the efficacy of suberin as a barrier.

In our previous study regarding the influence of storage conditions on the biochemical composition and morphology of tuberous *Dahlia* roots, several distinct zones were observed in the cross-sectional views of the tuberous roots [38].

In *Dahlia*, the primary root exhibits a tetra-protostele structure, characterized by four groups of liberin vessels alternating with primary wood, and a reduced pith [39]. The structure of the primary root can be described from exterior to interior as follows: rhizodermis, exodermis, cortex, and central cylinder. Within the central cylinder, the conducting vessel elements are arranged alternately in a radial pattern [40].

This investigation focused on the biochemical composition of tuberous root zones using FT-IR imaging to monitor variations across different stages of the vegetation cycle—early spring post-vegetative period; field planting; mid-vegetation in August; late fall. For the ‘Kennemerland’ cultivar, 25 spectra were collected from a defined section of the tuberous root measuring 50 µm × 50 µm. FT-IR mapping was subsequently performed to visualize the distribution of a specific spectral band across the entire scanned area. This approach used the area of the band instead of its position due to potential variations in band’s positions among spectra, which could lead to inaccuracies.

A critical consideration for understanding the changes in biochemical composition of tuberous roots pertains to the origin of the planting material (forced versus nonforced tuberous roots).

Determining the soluble dry matter in tuberous *Dahlia* roots using a Zeiss refractometer at the end of the vegetation period, researchers found a higher concentration of soluble dry matter (24.27%) for the ‘Kennemerland’ cultivar/nonforced tuberous roots compared to 16.43% for the ‘Kennemerland’ cultivar/forced tuberous roots [38]. Moreover, observations and biometric measurements revealed that forcing tuberous roots stimulates the plant to exhibit higher values for the studied traits, indicating that the dry matter found in tuberous roots is utilized by the plant for early entry into the vegetation period and throughout the vegetation period [38].

By analyzing FT-IR and FT-Raman spectra, it was demonstrated that inulin accumulation inside the tubers is favorably influenced by sand storage and depends on the cultivar type [38]. The current study establishes that tuberous root forcing affects *Dahlia* varieties. The results obtained from the analysis of the FT-IR spectra recorded on four sections of the tuberous *Dahlia* roots allowed the evaluation of the changes induced by the technological conditions applied to the biochemical composition; in addition, the vibrational imprint of inulin can be observed alongside other changes due to some active compounds induced by treatment before planting in the field.

The *Dahlia* roots examined under an optical microscope revealed that suberin-impregnated stacked cells could be identified toward the exterior, being produced by the phellogen. Suberin is a lipophilic macromolecule found in protective cells in plants [41,42]. Depolymerization of suberin releases 80–90% monomers such as aliphatic acids, 5–20% glycerol, and small amounts of phenolic acids such as ferulic acid [42]. For this reason, the band attributed to suberin was selected as a characteristic band of zone 1.

According to a study, wild *Dahlia* species are considered promising options for use as nutraceutical foods, contributing to health care [43]. The observed variability in these species makes them valuable candidates for selecting traits of nutraceutical interest for genetic improvement. Investigations concerning the chemical composition of some garden *Dahlia* tubers revealed that they are rich in carbohydrates, fiber, and proteins, in spite of the considerable variability observed among cultivars [44].

In a previous study, the experimental and theoretical Raman and FT-IR spectra of inulin were analyzed, and the assignment of different vibrational modes to the corresponding bands was performed [45]. Thus, it was found that the spectral region between 599 and 833 cm^−1^ in IR and Raman spectra is dominated by the bands assigned to the bending vibrations of various groups like C-C-C, O-C-O, and CH from different rings. Moreover, the most intense IR band at 1030 cm^−1^, with a shoulder at 1050 cm^−1^, and their corresponding Raman bands at 1028 cm^−1^ and 1050 cm^−1^ (calc. 1000, 1036 cm^−1^), respectively, proved to be due to the stretching vibration of the CO bond [45]. Thus, based on the findings from prior research [45,46], we identified in our spectra the characteristic absorption band of inulin at 936 cm^−1^.

In our study, the behavior of the bands specific to inulin, lignin, and suberin was utilized to highlight the differences that occur in tuberous roots during the vegetation period of *Dahlia*. The obtained results emphasized that these chemical compounds are present in the structure of tuberous roots throughout the vegetation period, but they vary depending on the analyzed root zone and the stage of the year in which the analysis was conducted.

## 4. Materials and Methods

### 4.1. Experimental Site and Pedoclimatic Conditions

These studies were conducted in Cluj-Napoca, Romania, in several locations, as follows: University of Agricultural Sciences and Veterinary Medicine of Cluj-Napoca: Didactic greenhouse of the Floriculture and Ornamental Arboriculture discipline: forcing of tuberous roots; Laboratory of Botany/Plant Morphology, Anatomy, and Plant Systematics: defining the zones in the cross-sections of *Dahlia* tuberous roots, Agrobotanical Garden: placement of the experimental field, Institute of Interdisciplinary Research in Bio-Nano-Sciences, Babeş-Bolyai University of Cluj-Napoca: FT-IR measurements.

The climate is generally influenced by the geographical location of the city, the set of relief conditions in which it is located, and its position in relation to the main components of the general circulation of the atmosphere.

From a climactic point of view, Cluj-Napoca has a moderate continental climate with oceanic influences. Temperature and precipitation can vary across the city’s regions due to the differences in the elevation ranges. The temperature is influenced by solar radiations, as well as dynamic and geographical factors [47]. Dahlias prefer well-drained soils with a medium texture that are rich in organic matter, especially those of the sandy-loam or loam-sand type [48,49]. *Dahlia* is an adaptable plant that thrives in rich chernozem soil but can grow in a variety of other soil types as well [50]. The most favorable soils must have a neutral pH [51]. When growing dahlias in pots, regulated and abundant flowering was observed at a pH of 5. However, the development of tuberous roots is better achieved at a pH of 6.5 [52].

The agrochemical and pedological characterization of the soil was conducted by obtaining eight soil samples from an experimental field. The soil analyses were performed on the composite sample collected from the experimental field at the Office for Pedological and Agrochemical Studies in Cluj-Napoca. During the experiments, the following parameters were determined and analyzed: soil pH; supply of mineral elements (nitrogen, phosphorus, potassium), soil moisture; humus content; and granulometric composition.

The results are presented in Table 2.

### 4.2. Plant Material

The biological material was purchased from the “Alexandru Borza” Botanical Garden, Cluj-Napoca, Romania.

*Dahlia* ‘Kennemerland’ can reach heights of up to 90–120 cm, characterized by herbaceous stems and grassy foliage. Its inflorescence is yellow, sterile, perfumed, and measures between 10 and 15 cm in diameter.

### 4.3. Analytical Methodology and Data Processing

In this study, experiments were conducted using both forced and nonforced tuberous roots of *Dahlia*. The forcing process took place in the educational greenhouse at the Faculty of Horticulture, UASVM Cluj-Napoca, and began in mid-March. The tuberous roots were planted in large pots filled with a planting medium composed of soil, peat, sand, and perlite in equal parts. Greenhouse temperatures were maintained at 18–20 °C, with air humidity levels at 70–80%.

This research was conducted as a bifactorial experiment organized in randomized blocks with three repetitions [53].

Two experimental variations emerged from combining experimental factors. Each variant had 15 tuberous roots, resulting in a total of 30 tuberous roots.

This study involved two main factors. Factor A was the planting material, which was available in two grades: a1, consisting of forced tuberous roots planted in the field, and a2, consisting of unforced tuberous roots planted directly in the field. Factor B was the cultivar, specifically b1, identified as ‘Kennemerland’.

Four regions (periderm, phloem, xylem, pith) were delineated in every cross-section of the tuberous roots during their examination under a Motic BA310POL microscope (MoticEurope, S.L.U. Barcelona, Spain), utilizing a 10× eyepiece and 40× objective.

Sections were taken from the four zones of the tuberous roots: zone 1 (periderm), zone 2 (phloem), zone 3 (xylem), and zone 4 (pith), from a total of 84 samples. These samples were utilized for FT-IR imaging measurements. The tuber regions, both forced and unforced, were scanned at various stages throughout the year, including during forcing, field planting, mid-season in August, and at the end of the season in fall.

Samples with thicknesses in the order of microns (20 µm) were sectioned from each tuberous root using a Nahita manual microtome model 501 (SC PRECISA SRL, Sibiu, Romania).

FT-IR spectra of tuberous roots were recorded using a Jasco IRT-5000 FT-IR microscope coupled to a Jasco FT-IR-6200 spectrometer (JASCO INTERNATIONAL Co., Ltd., Tokyo, Japan) with reflection configuration in the range of 800–4000 cm^−1^ and a resolution of 4 cm^−1^ using a 32× objective Cassegrainian, imaging a 50 × 50 µm sample area. Recorded spectra were smoothed using the 5-point Savitzky–Golay function. The FT-IR absorption spectrum of pure inulin powder (purchased from Alfa Aesar) (Thermo Scientific Chemicals, Ward Hill, MA, USA) was recorded with a JASCO 6200 spectrometer at room temperature in the spectral range of 400–4000 cm^−1^ with a resolution of 4 cm^−1^ using the pelleting technique with KBr.

The analyzed constituents of the tuberous *Dahlia* roots were inulin, lignin. and suberin. Over the course of the year, 84 sections were examined to observe the changes in these constituents. The spectra were processed using Spectra Manager^TM^ II software.

## 5. Conclusions

In conclusion, this FT-IR mapping of *Dahlia hybrida* tubers, focusing on key compounds such as inulin, lignin, and suberin across various growth stages and cultivation conditions, provides crucial insights into their spatial distribution and concentration. This detailed analysis not only illuminates how different cultivation practices and environmental factors impact the tubers’ biochemical makeup but also underscores their potential applications in the pharmaceutical, cosmetics, food, and dye extraction industries.

Inulin was the predominant constituent throughout the root surface. Following the evaluation of the mapping established by representing the ratios of the areas of some characteristic bands (inulin/lignin and inulin/suberin) for the ‘Kennemerland’ variety, the highest amount of inulin was found in zone 1 (autumn/forced and unforced tuberous roots, respectively) and in zone 2 (autumn unforced tuberous roots). Suberin was the constituent with the lowest concentration in tuberous *Dahlia* roots. The results found throughout this study revealed the fact that tuberous *Dahlia* roots contained a significant amount of inulin during the vegetation period, especially in spring. It turns out that by forcing, from spring to autumn, more inulin was consumed because early planting (forcing) caused the plant to start the assimilation processes earlier by developing a rich vegetative part early.

The findings underscore the significance of understanding how cultivation practices influence the composition of inulin, lignin, and suberin, pivotal components of tuberous *Dahlia* roots. This knowledge is essential for optimizing cultivation strategies to enhance yield, quality, and sustainability. Furthermore, it supports the development of new products and technologies across diverse industrial sectors.

While this study focused on the ‘Kennemerland’ cultivar in Cluj-Napoca, Romania, and acknowledges seasonal variations, it also highlights the need for broader spectroscopic investigations encompassing different *Dahlia* varieties and environmental contexts. Such research avenues are vital for enhancing the applicability and generalizability of findings in diverse settings and cultivars, thereby advancing our understanding and utilization of the tuberous roots of *Dahlia* in various industries.

## Figures and Tables

**Table 1:** Assignment of the main vibrational modes from FT-IR spectrum of tuberous roots of the cultivar ‘Kennemerland’.

IR Band	Vibrational Assignment
936 m	CO stretching (inulin)
1030 m	CO stretching (inulin)
1060 m	COC asymmetric stretching (cellulose)
1125 s	COC symmetric stretching (cellulose)
1130 s	CCO stretching + OH deformation + CH deformation (inulin)
1220 m/1240 m	CO stretching (lignin)
1330 m	CH and CH_2_ groups deformation (cellulose)
1430 s	CH and CH_2_ groups deformation (cellulose)
1600 s	C=C stretching (polyphenolic compounds)
1680 sh	amide I vibrations indicating the protein
1740 w	C=O groups stretching (suberin)
2930 s	CH stretching
3460 s broad	OH stretching

**Table 2:** Physicochemical properties of the soil in the experimental field.

Sample Identification		Name Analysis										
No. of Lab.	No. of Sample	pH	N %	Humus %	P mg L^−1^	K mg L^−1^	U %	Granulometric Analysis				
								Coarse Sand	Sand	Dust I	Dust II	Clay
572	Average	6.22	0.18	2.04	64	436	19.2	17.3	27.8	7.1	12.5	35.2

## Data Availability

Data are contained within the article.
